# Diagnosing an overcrowded emergency department from its Electronic Health Records

**DOI:** 10.1038/s41598-024-60888-9

**Published:** 2024-04-30

**Authors:** Luca Marzano, Adam S. Darwich, Raghothama Jayanth, Lethvall Sven, Nina Falk, Patrik Bodeby, Sebastiaan Meijer

**Affiliations:** 1 Department of Biomedical Engineering and Health Systems, KTH Royal Institute of Technology, Stockholm, Sweden; 2 Uppsala University Hospital, Uppsala, Sweden

**Keywords:** Health care, Health occupations, Medical research

## Abstract

Emergency department overcrowding is a complex problem that persists globally. Data of visits constitute an opportunity to understand its dynamics. However, the gap between the collected information and the real-life clinical processes, and the lack of a whole-system perspective, still constitute a relevant limitation. An analytical pipeline was developed to analyse one-year of production data following the patients that came from the ED (n = 49,938) at Uppsala University Hospital (Uppsala, Sweden) by involving clinical experts in all the steps of the analysis. The key internal issues to the ED were the high volume of generic or non-specific diagnoses from non-urgent visits, and the delayed decision regarding hospital admission caused by several imaging assessments and lack of hospital beds. Furthermore, the external pressure of high frequent re-visits of geriatric, psychiatric, and patients with unspecified diagnoses dramatically contributed to the overcrowding. Our work demonstrates that through analysis of production data of the ED patient flow and participation of clinical experts in the pipeline, it was possible to identify systemic issues and directions for solutions. A critical factor was to take a whole systems perspective, as it opened the scope to the boundary effects of inflow and outflow in the whole healthcare system.

## Introduction

Emergency departments (EDs) are essential components in healthcare systems by providing critical care to patients requiring immediate medical attention^1^. ED overcrowding is characterized by an increased number of patients seeking care, resulting in long wait times, treatment delays, and reduced quality of care^2–5^.

This problem persists globally^1,6^ despite the differences between healthcare policies in different countries^7,8^ , Sweden being no exception^6,9–11^. Previous studies showed a high workload for the main Swedish hospitals^12^, pointing out the multifaced nature of operational errors^11,13^, negative patient experience of high waiting times^14^, and the decreasing availability of beds followed by an increasing of patients visiting ED^6^.

This problem is challenging because of the complexity of the system operations and diversity of clinical profiles of the patients^15,16^. Indeed, a high volume of patients visiting EDs corresponds to a wide range of medical conditions, from patients that need basic care to those with an urgent need for intervention due to the severity of the conditions, with a constrained number of resources to treat them often subjected to cost pressures^16,17^.

In recent years, the use of real-world data in clinical practice to inform clinical decisions and systems operations has attracted significant interest^18–20^. Healthcare production data and Electronic Health Records (EHRs) present an opportunity to comprehensively analyse ED overcrowding and enhance healthcare system operations and management^19–21^.

Several techniques to exploit real-world data have been proposed and discussed to address the challenge of ED overcrowding in operational research^21,22^. These techniques span from traditional approaches such as multivariate linear models^22^ and simulation process modelling^23,24^, to novel techniques based on machine learning^25,26^ and process mining^27,28^.

Most data-driven approaches retrospectively analyse the data to explore, explain and predict operational variables, such as admissions, re-visits, triage, diagnosis, and length of stay^16,25,29–38^. Simulation studies have been used for the purpose of performance evaluation and testing layout planning^39–42^ with a focus on the optimization of scheduling management^43,44^. Process mining has been applied for the extraction of clinical pathways directly from EHRs^45^ to improve capacity management^46^ and to cluster patient trajectories based on similar clinical characteristics^47,48^. Few participatory approaches involving experts have been used to investigate this problem from the perspective of the different actors involved (e.g., explore the possibility to use past medical records to inform admission decisions, and study of re-visits through created personas from the data records^49–52^) and dashboard development to visualize key performance indicators (KPIs) in real-time^53,54^.

However, the gap between real-world data and the actual processes that occur in emergency departments constitutes a key limitation^29,35,55^. Indeed, the gap between real operations and abstraction made from event log data is considered a substantial challenge^56,57^. This not only limits the effectiveness of pure data-driven approaches but also affects the simulation and process mining approaches^27,58,59^. Moreover, the reliability of data-driven approaches is limited by the discrepancies between real-world data primary users and collected information from the clinical experts^35^.

Previous works mainly refer to supporting better operational decisions^43^, often attempting to optimise a single key performance indicator (KPI) or specific flows treating the ED as an isolated system^60^, but with limited focus on the policy-level analysis to solve the overcrowding problem^41,61^. Moreover, the focus of previous data-driven analysis has been on the volume of flows rather than clinical variability^16,33,62–65^, missing considerations on how the complexity of medical evaluation can impact prompt decisions^16,17,34^.

Despite the large amount of published works and variety of approaches, further research is still necessary to understand the potential of healthcare data for informing reduction in overcrowding and enhance the quality of care in the ED. In fact, to study the complexity and the multi-constrained nature of the overcrowding makes necessary to consider the effect of processes happening outside the ED^41^. For example, the efficiency of ED discharge could be affected by the delay of hospital admission due to overcrowding of the wards, the so-called boarding^66^, or further pressure can originate from factors outside the hospital^67^.

The involvement of experts in the analytical process is necessary to leverage these challenges, increase the understanding of phenomena beyond the real-data limitations, and explore future design strategies^68^. Hence, a whole-system approach is required to develop reliable solutions for practical applications^15,34,69^.

To summarise, there is a need to develop approaches that go beyond pure empirical approaches to leverage real-world data to address ED overcrowding. Therefore, we aimed to develop a pipeline to analyse ED data from a whole-system perspective that strives to overcome the limitations of the data information and discuss deeply causes and potential solutions of the overcrowding. The ED whole-system perspective is given by involving clinical experts in all the analysis steps and integrating external data or information that is not collected in the ED data regarding the admitting wards and the processes happening outside the hospital.

This pipeline was designed to analyse a real-world case study that consisted of one year (2019) of hospital production data following patients that visited the Uppsala University Hospital ED. The Uppsala ED constituted an ideal case study because of the reported serious shortcomings and hospital overcrowding in the timespan of the data records^6,9–11^.

### Hospital emergency department production data

The Uppsala University Hospital’s (Sweden) ED production data from 2019 were analysed (n = 33,881 patients for n = 49,938 total event logs). It is the only emergency department in Uppsala city and the largest in the Uppsala county, and it operates 24 h with two main access points: directly from the ambulance entrance, or through a walk-in reception. Previously, these data were used to inform a simulation study aimed to improve the ED acute flows testing which kind of interventions the hospital needed to reach a 4-h length of stay target^70^.

In Tables 1, 2 we reported the summary of the cohort. The following variables were included for each record: age, sex, ADAPT triage code^71^ (red: “life-threatening”, orange: “seriously ill”, yellow: “ill”, green: “need of assessment”, blue: “minor injuries or illnesses that can be quickly treated and discharged”, and white: “no need of urgent care or monitoring”), chief complaint reason for the visit, arrival with ambulance (y/n), imaging scan (y/n), main diagnosis in ICD10 codes (https://icd.who.int/browse10/2019/en ), waiting time (from arrival to first contact) and length of stay (from arrival to discharge in the ED), the reason for discharge (sent home, admitted to a hospital ward, death, or other reasons). The ward for each admitted patient to the hospital was also reported. Moreover, eventual reasons of the ED visit (e.g., referral) and specific method of arrival if not from ambulance (e.g., pedestrian, or special transport from geriatric or psychiatric facilities) were retrieved (Supplementary Table 1).

**Table 1:** Summary of the cohort data.

	Method of discharge			
	Other reasons	sent home	admitted to hospital	Overall
	(N = 6183)	(N = 30,773)	(N = 12,982)	(N = 49,938)
Sex				
Female	3246 (52.5%)	15,776 (51.3%)	6413 (49.4%)	25,435 (50.9%)
Male	2937 (47.5%)	14,996 (48.7%)	6569 (50.6%)	24,502 (49.1%)
Missing	0 (0%)	1 (0.0%)	0 (0%)	1 (0.0%)
Age (years)				
Median [Min, Max]	59.0 [0, 102]	51.0 [0, 104]	73.0 [1.00, 104]	58.0 [0, 104]
Missing	1 (0.0%)	8 (0.0%)	1 (0.0%)	10 (0.0%)
Ambulance				
No	3850 (62.3%)	24,946 (81.1%)	6198 (47.7%)	34,994 (70.1%)
Yes	2333 (37.7%)	5827 (18.9%)	6784 (52.3%)	14,944 (29.9%)
Triage				
Yellow	2349 (38.0%)	11,807 (38.4%)	5370 (41.4%)	19,526 (39.1%)
White	1121 (18.1%)	6155 (20.0%)	2875 (22.1%)	10,151 (20.3%)
Green	946 (15.3%)	7612 (24.7%)	1049 (8.1%)	9607 (19.2%)
Orange	734 (11.9%)	1543 (5.0%)	1916 (14.8%)	4193 (8.4%)
Red	61 (1.0%)	73 (0.2%)	266 (2.0%)	400 (0.8%)
Blue	9 (0.1%)	40 (0.1%)	6 (0.0%)	55 (0.1%)
Missing	963 (15.6%)	3543 (11.5%)	1500 (11.6%)	6006 (12.0%)
Chief complaint (reason for visit)				
Other	3266 (52.8%)	14,006 (45.5%)	4253 (32.8%)	21,525 (43.1%)
Abdominal pain	986 (15.9%)	4361 (14.2%)	2065 (15.9%)	7412 (14.8%)
Chest pain	418 (6.8%)	3537 (11.5%)	1097 (8.5%)	5052 (10.1%)
Difficulty breathing	337 (5.5%)	1355 (4.4%)	1537 (11.8%)	3229 (6.5%)
Leg swelling / pain	234 (3.8%)	2356 (7.7%)	281 (2.2%)	2871 (5.7%)
Neurological disorders	205 (3.3%)	1048 (3.4%)	1125 (8.7%)	2378 (4.8%)
General weakness	257 (4.2%)	589 (1.9%)	921 (7.1%)	1767 (3.5%)
Arrhythmia	100 (1.6%)	983 (3.2%)	498 (3.8%)	1581 (3.2%)
Back pain	106 (1.7%)	1126 (3.7%)	229 (1.8%)	1461 (2.9%)
Dizziness	195 (3.2%)	862 (2.8%)	327 (2.5%)	1384 (2.8%)
Hip injury	79 (1.3%)	550 (1.8%)	649 (5.0%)	1278 (2.6%)
Imaging evaluation				
No	4257 (68.9%)	21,095 (68.6%)	6340 (48.8%)	31,692 (63.5%)
Yes	1926 (31.2%)	9678 (31.4%)	6642 (51.2%)	18,246 (36.5%)

We grouped the less frequent levels of chief complaint and ICD10 codes due to the large number of levels. “Other reasons od discharge” indicated patients died in ED or deviated to another special services/consultant. SD: “standard deviation”.

**Table 2:** Summary of the cohort data.

	Method of discharge			
	Other reasons	sent home	admitted to hospital	Overall
	(N = 6183)	(N = 30,773)	(N = 12,982)	(N = 49,938)
First diagnosis ICD10 group				
R generic symptoms	2352 (38.0%)	13,215 (42.9%)	6119 (47.1%)	21,686 (43.4%)
Other	2168 (35.1%)	5698 (18.5%)	2449 (18.9%)	10,315 (20.7%)
S fractures	733 (11.9%)	5632 (18.3%)	1269 (9.8%)	7634 (15.3%)
M muscular and connective tissue	231 (3.7%)	3353 (10.9%)	424 (3.3%)	4008 (8.0%)
K gastrology	319 (5.2%)	1452 (4.7%)	1302 (10.0%)	3073 (6.2%)
I circulatory apparatus	262 (4.2%)	1304 (4.2%)	1369 (10.5%)	2935 (5.9%)
Missing	118 (1.9%)	119 (0.4%)	50 (0.4%)	287 (0.6%)
First diagnosis ICD10 code				
Other	3177 (51.4%)	19,216 (62.4%)	7942 (61.2%)	30,335 (60.7%)
R104X	603 (9.8%)	3221 (10.5%)	862 (6.6%)	4686 (9.4%)
R074	133 (2.2%)	2123 (6.9%)	602 (4.6%)	2858 (5.7%)
R060	119 (1.9%)	632 (2.1%)	800 (6.2%)	1551 (3.1%)
R429	136 (2.2%)	752 (2.4%)	245 (1.9%)	1133 (2.3%)
Z711	935 (15.1%)	163 (0.5%)	1 (0.0%)	1099 (2.2%)
R539	129 (2.1%)	360 (1.2%)	598 (4.6%)	1087 (2.2%)
R519	91 (1.5%)	817 (2.7%)	97 (0.7%)	1005 (2.0%)
R559	263 (4.3%)	399 (1.3%)	276 (2.1%)	938 (1.9%)
R298	59 (1.0%)	260 (0.8%)	617 (4.8%)	936 (1.9%)
R224	50 (0.8%)	745 (2.4%)	51 (0.4%)	846 (1.7%)
R529	51 (0.8%)	593 (1.9%)	100 (0.8%)	744 (1.5%)
M549	66 (1.1%)	527 (1.7%)	110 (0.8%)	703 (1.4%)
R509	188 (3.0%)	113 (0.4%)	350 (2.7%)	651 (1.3%)
Missing	118 (1.9%)	119 (0.4%)	50 (0.4%)	287 (0.6%)
Hospital admission ward				
Other	1071 (17.3%)	0 (0%)	5464 (42.1%)	6535 (13.1%)
Acute medicine ward	113 (1.8%)	0 (0%)	2163 (16.7%)	2276 (4.6%)
Cardiology ward	69 (1.1%)	0 (0%)	1996 (15.4%)	2065 (4.1%)
Surgery ward	97 (1.6%)	0 (0%)	1776 (13.7%)	1873 (3.8%)
Orthopedic ward	94 (1.5%)	0 (0%)	1341 (10.3%)	1435 (2.9%)
Missing	4739 (76.6%)	30,773 (100%)	242 (1.9%)	35,754 (71.6%)
Waiting time (hours)				
Median [Min, Max]	1.01 [0.000278, 13.0]	1.37 [0.000556, 25.4]	1.01 [0.00111, 14.8]	1.23 [0.000278, 25.4]
Missing	1078 (17.4%)	896 (2.9%)	368 (2.8%)	2342 (4.7%)
Length of stay in the ED (hours)				
Median [Min, Max]	4.72 [0.0181, 28.9]	4.35 [0.0169, 49.2]	6.22 [0.0544, 52.4]	4.79 [0.0169, 52.4]

“Other reasons od discharge” indicated patients died in ED or deviated to another special services/consultant. SD: “standard deviation”. ICD10 texts: R104X Abdominal pain, unspecified; R074 Chest pain, unspecified; R060 Dyspnoea; R429 Dizziness and vertigo; Z711 Person with suspected disease where no diagnosis is made; R539 general weakness feeling sick and tired; R519 Headache; R559 Fainting and collapse; R298 Other and unspecified symptoms and signs of disease of the nervous system and musculoskeletal system; R224 Localized swelling or lump of lower extremity; R529 Pain or ache, unspecified; M549 Back pain, unspecified; R509 Fever, unspecified; I489 Atrial fibrillation and atrial flutter, unspecified; M796G Pain, nonspecific in lower leg.

During the analysis the hospital records regarding number of assigned patients and available beds for each hospital ward were also available. In Supplementary Table 2 we reported the summary of the patients admitted in the hospital stratified by speciality of the ward. Here we also reported how many patients were allocated in the right ward. This information was possible to retrieve with the aid of the clinical experts by looking through the medical alarm unit of the Uppsala internal system associated to the admitted patients and compare that with the speciality of the ward. According to the clinical experts, this information was relevant to study because wrong admissions are usually correlated to the lack of available places in the right wards.

## Methods

### Ethics declaration

The ethical approval regarding the usage of the data with the purpose of the presented research was approved by Uppsala University Hospital (case number: FOU2024-00,078). The need for informed consent was waived by Uppsala University Hospital. The entire research was performed in conformance with the WMA Declaration of Helsinki.

### Analytical pipeline

**Figure 1:**
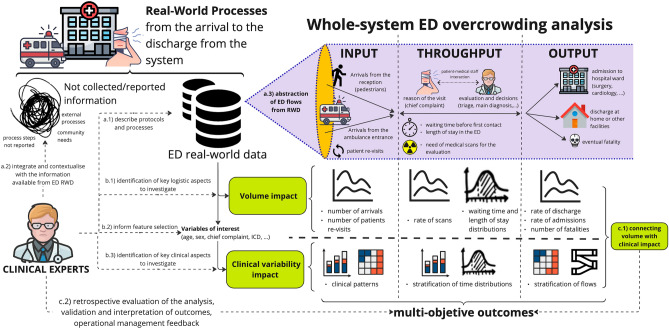
Real-world data pipeline. Prior to commencing the analysis, the clinical experts explained data in function of processes and protocols (a.1), and integrated information not available from the data (a.2). The flow characteristics are abstracted from the data by dividing it into three components (a.3): input, throughput, and output flow. Then, clinicians identify key logistics and clinical aspects to investigate, and they inform the feature selection (b.1–3). Finally, the series of outcomes are evaluated by a validation and interpretation process followed by a discussion regarding overcrowding and improvement of operations (c.1–2).

In Fig. 1 is reported the analytical pipeline. Prior to commencing the analysis, the clinical experts described the processes and protocols behind the ED data (step a.1). All data analyses were carried out using RStudio, version 2022.12.0 + 353. RStudio was also used to create the plots presented in the results. A relevant passage was the integration of information regarding external factors that influence the ED performances but that are not collected in the data records (step a.2). These include additional information at higher level of granularity of the real process, and external factors such as patients coming from special facilities and community needs (e.g., psychiatric, and geriatric care).

After contextualizing and explaining the variables, we abstracted the flow characteristics from the data by dividing it into three components (step a.3): input, throughput, and output flow. This follows previously proposed approaches to model ED flows and categorize interventions into types^72^ and associated key performance indicators (KPIs)^60^.

Once the description of the flow from arrival to discharge from ED is abstracted from the data, the impact of patient volume on ED functioning was studied (step b.1). This was done by detecting the possible KPIs that can be computed from the records. Each KPI was computed in relation of the abstraction component to which it belongs. Time series were deployed for the study of the daily metrics, and aggregated statistics distribution for the hourly and absolute values.

The detected KPIs were associated to the flow components in the following way:

- Input: number of arrivals, total and with the ambulance, and patient re-visits;
- Throughput: rate of performed imaging assessment and time distributions for waiting time and length of stay;
- Output: rate of discharges, admissions to the hospital, and number of fatalities.

Following the volume analysis, we investigated how KPIs are connected to the clinical variability of the patients (step b.2 and b.3). In this part of the framework the patient-based variables (e.g., age, sex, chief complaint) were explored in connection with the ones obtained by clinical decisions (e.g., triage, ICD10 diagnosis, scans, and final discharge/admission decision). For the input KPIs the clinical patterns were explored with aggregate statistics and stratification of time series. Special attention was given to the chief complaint and ICD 10 diagnosis by designing an interaction matrix between these two variables to assess the variability of clinical decisions. Single patient re-visits were studied by mining chief complaint-ICD10 sequences from each visit to study patterns and longitudinal correlations between the previous visits.

For the throughput KPIs the time distributions were stratified in function of the clinical variables, and multiple variables were studied by heatmaps referring to the metrics. This allowed to explore if there were operational bottlenecks or patterns in patients with long waiting or length of stay in the ED. A multivariate linear regression was performed as preliminary assessment of the association between length of stay and the variables.

Output flow was analysed in concomitance with input flow component. Furthermore, Sankey flows were adopted to picture the variability of the variables in function of the time moment in flow, thus connecting the input-throughput variability with the final decision. The final decision, including special structures of admission to a ward to the hospital, were considered in this stage to make considerations from the ED to a whole-system perspective. During this passage the information regarding the hospital ward availability was integrated.

Clinical experts informed the analysis of patient volume and clinical variability by identifying logistic and clinical aspects to investigate, including the feature selection of variables of interest to connect volume KPIs with the clinical characteristics of the patients. Finally, clinical experts were involved to evaluate, validate, and interpret the series of outcomes obtained from the pipeline (step c.1 and c.2). This step included a discussion on the operational management aspects of overcrowding and possible future interventions (step c.2).

In this work special, attention was given to the interaction between chief complaint and ICD-10 code of the first diagnosis since these two variables were representative of the interaction between patient and ED practitioners ‘decision. For what concerns the volume and clinical variability analysis, the main investigation was regarding how to stratify the ED flows. Clinicians suggested to consider four main stratifications: patients with need of urgent care, patients with non-urgent need of care and simple to process (“see and treat”), patients requiring complex examination in the ED from which there would be a competitive decision between discharge or send to an hospital ward, and geriatric patients that need basic care. The geriatric flow was the one connected to external processes to the hospital that concerned mostly the clinicians. The urgent care flow management in competition with non-urgent and complex patients was studied in the previous simulation work^70^.

## Results

**Figure 2:**
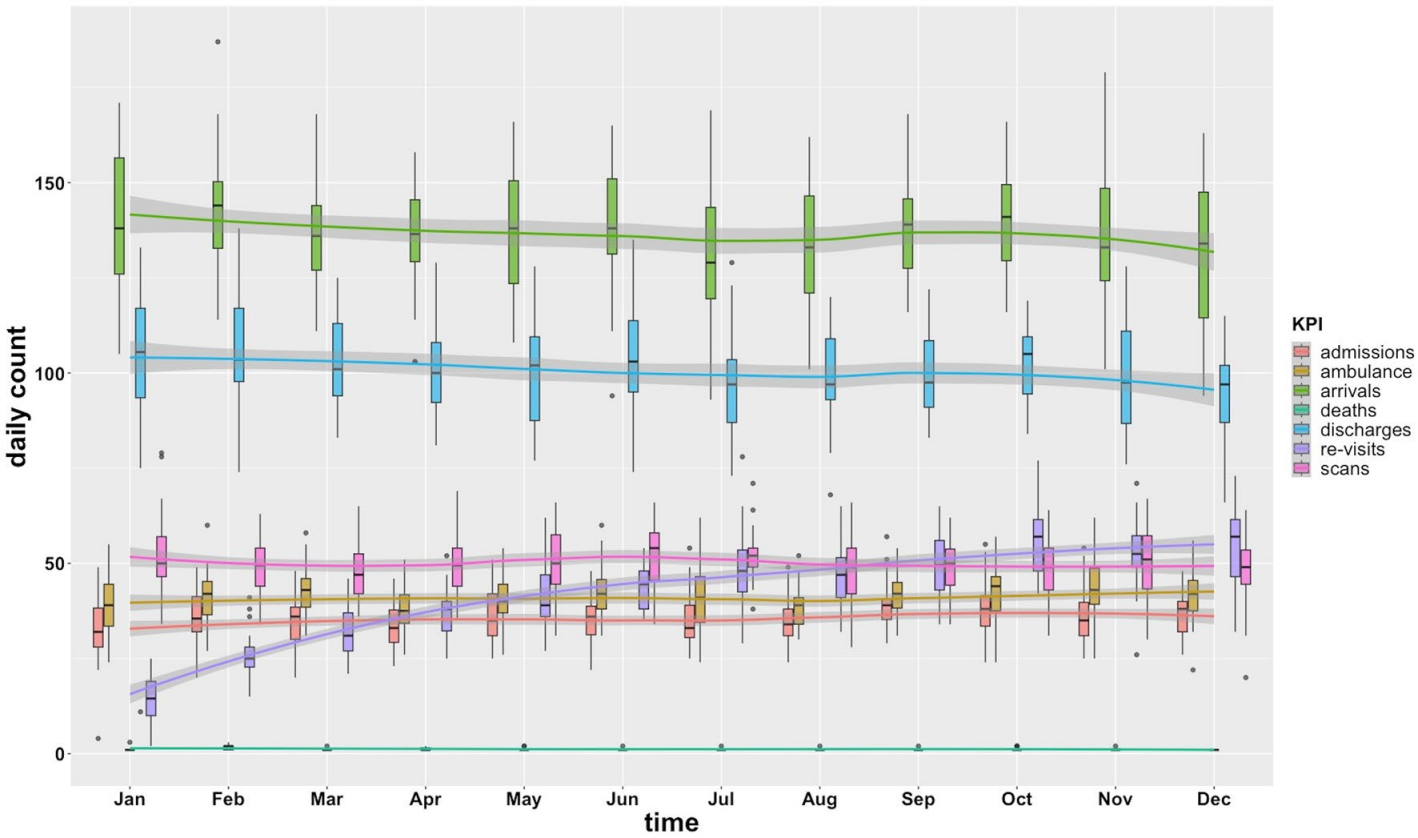
Volume impact of KPIs during the year. The trend lines refer to the daily count obtained with a local polynomial regression fit. Boxplots refers to the daily count distribution of each month.

In Table 3 we reported a summary of the key results with the associated feedback of clinicians, and the potential research for future intervention. In Fig. 2 instead the KPIs daily impact along the year are plotted.

**Table 3:** Key results regarding the main sources of Uppsala ED overcrowding followed by clinical feedback and future research perspectives.

key results (Main flow component)	Reference	Clinicians’ feedback	Future research
Majority of ED patients are classified as patients that do not need high urgent care (Input)	Table 1, Figs. 3, 5	Over-usage of ED also from patients that are not supposed to be there The data provided a magnitude and level of scale of the problem There is the need to stratify and separate the different flows	Detect and track patients that are not supposed to visit ED for seeking care Explore if this pressure could have been generated by pitfalls of primary care delivery
First evaluation ICD10 code is mainly generic symptoms or non-specific diagnosis. Moreover, there is a key bulk of patients with feared health complaint in whom no diagnosis is made (Input)	Table 2	Expected result regarding generic symptoms patients. High presence of non-specific diagnosis was not expected Z711 patients are impacting more than expected -difficulties to group ICD10 codes in function of logistic operations from the data	Discussion on how to improve accuracy of the first evaluation of patients to make the decision process faster Discussion on the current variables and standard (such as ICD10 code) are capable to capture the right level of clinical detail
The main source of clinical variability is from patients diagnosed with generic symptoms (Input)	Figures 3, 5, Supplementary Fig. 1	This result was interestingly surprising because it was expected to see different patterns by stratifying the variables It would be challenging stratify the patients in the defined sub-flows from this data	Discussion on how to improve the collected information to detect and separate properly sub-flows of patients
Patients spend long times in the ED regardless their triage, chief complaint, or first diagnosis: a clear symptom of the ED saturation (Throughput)	Tables 1, 2, Fig. 4, Supplementary Fig. 2	High waiting time and length of stay because these were problems, we were a priori aware of Some outliers correspond to highly fragile elderly patient not living in special facilities The relationship between imaging and admission is connected to the sub-flow of patients difficult to evaluate The common high distributions and presence of outliers is pointing out that ED is saturated	Discussion on the current length of stay targets (2–4 h) and the feasibility to reach them Discussion on the validity of approaches and models developed from data of saturated systems like the ED departments Local analysis outlier by outlier to explore further patterns or new sub-flows
High number of requests for imaging assessment during ED visits. Length of stay for these patients is wider and longer compared to other patients (Throughput)	Tables 1, 2, Figs. 2, 4	High number of scans was an expected result Further insights regarding the high magnitude and the delay of decisions of patients difficult to evaluate An over-request of imaging assessment can be related to the decisions made by less experienced doctors (e.g., junior doctors) It is challenging to separate complex from “see and treat” patients from the current data	Discussion on how to improve the evaluation process of patients with difficult or not clear clinical conditions. This also includes the interaction between senior and junior doctors
A relevant number of patients with generic symptoms, often with advanced age, is admitted to all hospital wards. All the hospital wards resulted overcrowded and with patients not supposed to be admitted there (Output)	Figure 5, Table 4, Supplementary Fig. 1, Supplementary Figs. 3, 4, Supplementary Tables 2, 3, 4	Expected result to see the association between admission and higher age Patients with generic symptoms can be admitted everywhere was a new insight. It was only expected the impact of abdominal pain patients to the surgery teams The analysis underlined the key role of the boarding effect from the hospital wards and the wrong allocation of patients	It is required to study deeper the boarding problem, and how overcrowding between ED and hospital wards interaction Deeper analysis of generic symptoms patients that need to be admitted in the hospital
Patients re-visiting often the ED are impacting globally and significantly to the overcrowding (Output, Input)	Figure 2, Supplementary Table 5	We were expecting to see the re-visits of geriatric and psychiatric patients, while generic symptoms repeated visits were not accounted before New insight regarding how re-visits are impacting on all the aspects related to the overcrowding, thus affecting all the ED efficiency Interesting insight regards the fact that few patients that are revisiting often the ED can provide a resonant impact on the global system	A deeper analysis of these highly frequent patients. Exploring the possibility to retrieve more information about these patients Geriatric and psychiatric flow deviation would decrease the ED pressure Improve the evaluation to provide more accurate and prompt diagnosis to avoid re-visits. Finding a way to predict or track these high frequent patients would allow to a better management of the global ED pressure

### Non-urgent patients and generic or non-specific diagnosis

**Figure 3:**
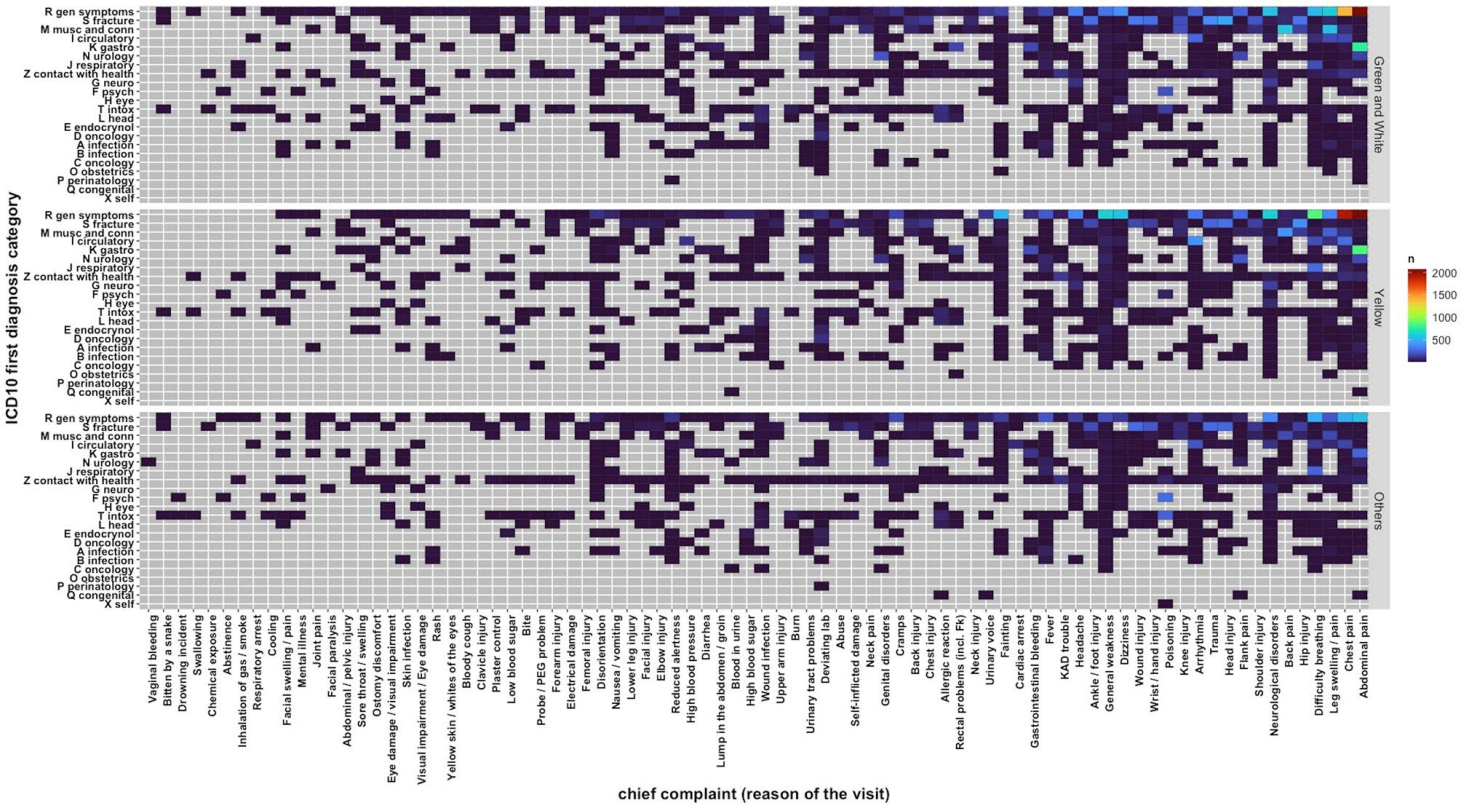
Heatmap of the absolute occurrence of chief complaint and main ICD10 category group of the first diagnosis during the year stratified by triage code. Green and white triage were grouped to quantify the impact of extremely low urgent patients. “Others” category grouped blue, red, and missing triage visits due to the lower frequency compared to the other categories. Heatmap created using the ggplot library in RStudio.

Most patients that visited ED in 2019 were patients having not urgent care: 39.1% triage yellow code, 20.3% white, and 19.2% green on the total visits (Table 1). 82.2% of pedestrians visited the ED without a referral (Supplementary Table 1). Figure 3 shows the heatmap of the yearly reported chief complaint and ICD10 main diagnosis stratified by triage to capture the magnitude of clinical variability as a function of the interaction patient-clinician (patient: chief complain—clinician: ICD10 diagnosis). This plot shows how heterogenous is the clinical information regardless urgency of care, and that from any reason of the ED visit their main diagnosis can fall in any kind of ICD10 category. This could be deduced by the fact that the majority of defined cluster of patients, such as abdominal and chest pain (n = 12,464; 24.9%), are diagnosed with the redundant ICD10 referring to the generic symptom (R104X “Abdominal Pain, unspecified” and R074 “Chest pain, unspecified”). Except for some group of patients with defined categories, such as patients having fractures or cardiovascular diseases, it becomes hard to identify more specific categorizations from the data.

Another surprising aspect related to the main diagnosis can be discovered if we look to the most frequent ICD10 diagnosis complete codes (Table 2). Most of the diagnoses was from the generic symptoms category (ICD10 group R), but surprisingly also most of other codes from the other ICD10 groups resulted in non-specific diagnoses (e.g., M549 “Back pain, unspecified”, I489 “Atrial fibrillation and atrial flutter, unspecified”, M798G “Pain, nonspecific in lower leg”, and N390 “urinary tract infection, site not specified”). Interestingly, patients with Z711 code diagnosis (“feared health complaint in whom no diagnosis is made”), patients that do not need urgent care from ED, were the second most common diagnosis after generic symptoms.

### Length of stay underlines the saturation of the ED

**Figure 4:**
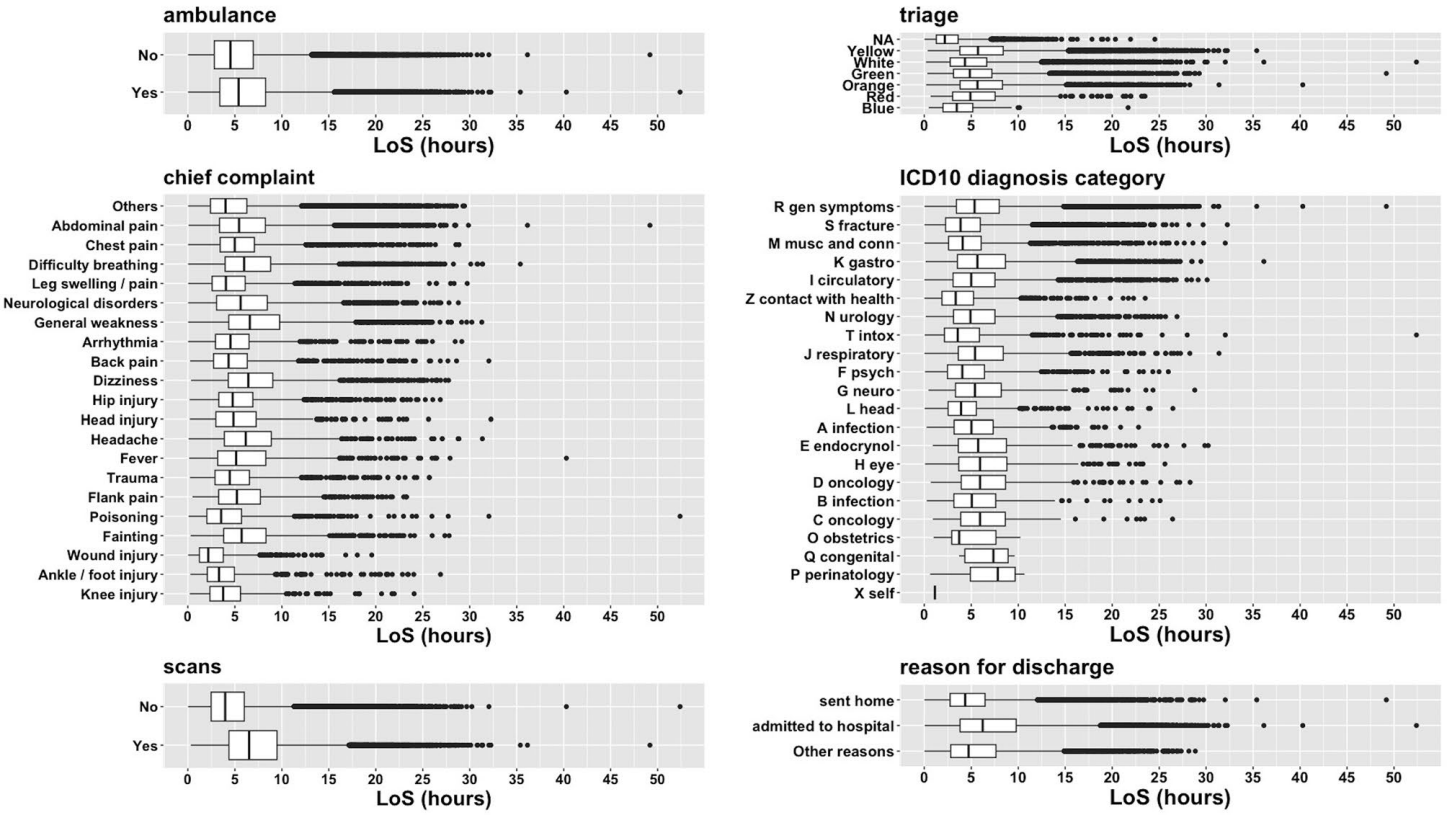
Length of stay distribution stratified by arrival with ambulance, triage, chief complaint, ICD10 category group, scans, and reason for discharge. The labels are sorted by decreasing frequency.

The length of stay was long with a large variability (Mean ± Standard deviation: 5.79 ± 4.21 h). Regardless of triage, chief complaint, or ICD-10 category, the length of stay was similar, with a high number of outliers of long staying in the ED for any category (Fig. 4). As expected, some partial differences were detected stratifying waiting time by triage, but long waiting time and outliers were associated also to patients with urgent care codes, thus showing similar patterns of the length of stay distributions.

As shown in Fig. 4, length of stay of patients for which imaging assessment was requested (6.51 ± 4.81 h) was clearly wider and higher compared to the patients that were not (3,96 ± 3,39 h). According to the clinical experts, the number of scans performed in the ED (Fig. 2) is currently extremely high, and the possible causes could rely on not necessary imaging assessment requested by doctors with premature experience when evaluating patients with complex clinical profiles.

The multivariate regression confirmed the high impact of scans on length of stay and detected as relevant the reason for discharge and the age (See Supplementary Results). However, the R-square coefficient underlined that the linear assumption for length of stay was not captured by the data (R = 0.26). This confirms that the saturation of the ED reflected in the data makes multivariate predictions of the length of stay challenging based on the data.

### Effects of the overcrowded wards to the ED efficiency

**Figure 5:**
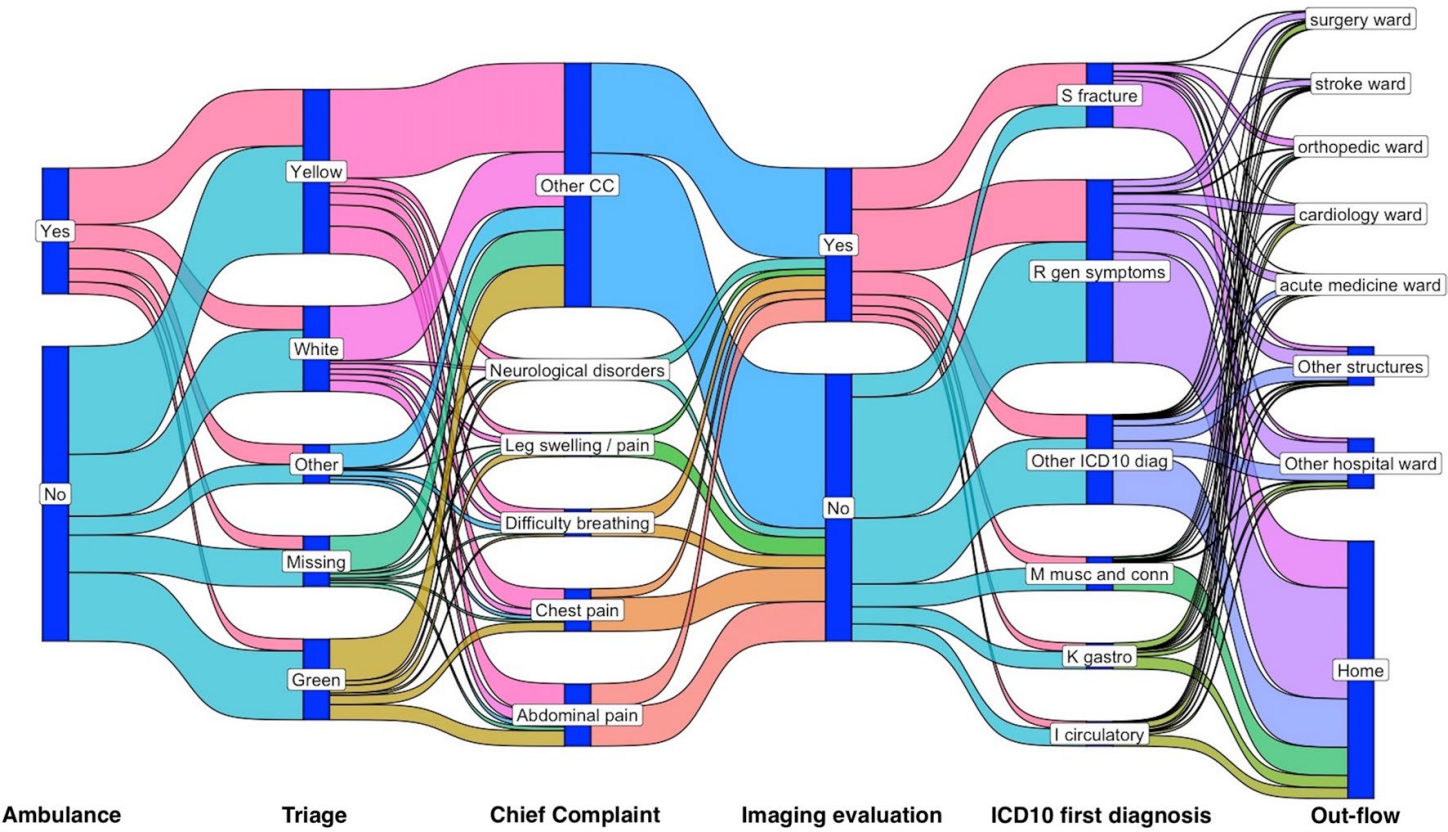
Sankey flow of the data variables. The displayed order of the flow corresponds to the chronological occurrence of the variable during the decision process. Due to the high number of levels of chief complaint, ICD10 category, and out-flow wards, the less frequent levels were grouped in one category.

The most common hospital admissions from ED were to the surgery, acute medicine, orthopaedic, cardiology, and stroke wards (Table 2 and Fig. 5).

According to the hospital records, all these wards were overcrowded during the entire the year, thus showing the probable effect of the hospital boarding on increasing the length of stay for ED patients waiting for an available bed in the ward. This can be seen in Table 4 where the most frequent admitting wards (almost all days of 2019) are reported with the daily admissions from the ED and the actual availability of the ward represented by the difference between total number of patients assigned and the number of beds. In Supplementary Table 3 we reported the same information in Table 4 for all the wards.

**Table 4:** The most frequent admitting wards of ED patients.

Speciality ward (hospital building), D = number of days when there were admissions from the ED	Number of daily admissions from ED	N total daily number of available places in the ward	M total daily patients of the hospital assigned to the ward	Difference between available places N and number of allocated patients M
Acute medicine ward ( Medicinavdelning 30 E )	:	:	:	:
(D = 358)	Mean (SD): 3.72 (1.86)	Mean (SD): 20.7 (1.93)	Mean (SD): 24.0 (2.61)	Mean (SD): -3.33 (1.62)
	Median [Min, Max]: 4.00 [1.00, 11.0]	Median [Min, Max]: 21.0 [16.0, 26.0]	Median [Min, Max]: 24.0 [18.0, 32.0]	Median [Min, Max]: -3.00 [-9.00, 0]
		Missing: 0 (0%)	Missing: 0 (0%)	Missing: 0 (0%)
Cardiology ward ( Hjärtavdelning 50 F )	:	:	:	:
(D = 351)	Mean (SD): 3.32 (1.74)	Mean (SD): 24.5 (2.45)	Mean (SD): 27.2 (2.45)	Mean (SD): -2.72 (2.08)
	Median [Min, Max]: 3.00 [1.00, 8.00]	Median [Min, Max]: 25.0 [21.0, 31.0]	Median [Min, Max]: 27.0 [20.0, 33.5]	Median [Min, Max]: -3.00 [-9.00, 3.00]
		Missing: 0 (0%)	Missing: 0 (0%)	Missing: 0 (0%)
Stroke ward ( Strokeavdelning 85 B )	:	:	:	:
(D = 347)	Mean (SD): 2.98 (1.52)	Mean (SD): 19.5 (2.61)	Mean (SD): 19.9 (2.05)	Mean (SD): -0.401 (2.07)
	Median [Min, Max]: 3.00 [1.00, 8.00]	Median [Min, Max]: 19.0 [16.0, 23.0]	Median [Min, Max]: 20.0 [14.0, 25.0]	Median [Min, Max]: -0.500 [-7.00, 6.00]
		Missing: 0 (0%)	Missing: 0 (0%)	Missing: 0 (0%)
Orthopedic ward ( Ortopedavdelning 70 A )	:	:	:	:
(D = 342)	Mean (SD): 2.86 (1.50)	Mean (SD): 22.9 (2.33)	Mean (SD): 24.4 (1.90)	Mean (SD): -1.53 (1.57)
	Median [Min, Max]: 3.00 [1.00, 8.00]	Median [Min, Max]: 24.0 [18.0, 24.0]	Median [Min, Max]: 24.0 [18.5, 28.0]	Median [Min, Max]: -1.50 [-8.00, 2.00]
		Missing: 0 (0%)	Missing: 0 (0%)	Missing: 0 (0%)
Surgery ward ( Kirurgavdelning 70 E )	:	:	:	:
(D = 341)	Mean (SD): 3.32 (1.76)	Mean (SD): 21.7 (3.66)	Mean (SD): 27.2 (3.32)	Mean (SD): -5.46 (2.51)
	Median [Min, Max]: 3.00 [1.00, 9.00]	Median [Min, Max]: 20.0 [20.0, 30.0]	Median [Min, Max]: 26.0 [21.0, 37.0]	Median [Min, Max]: -5.50 [-13.5, 4.00]
		Missing: 0 (0%)	Missing: 0 (0%)	Missing: 0 (0%)
Acute medicine ward ( Akutvårdsavdelning 30 C )	:	:	:	:
(D = 325)	Mean (SD): 2.56 (1.41)	Mean (SD): 14.2 (1.40)	Mean (SD): 15.2 (1.56)	Mean (SD): -1.01 (1.01)
	Median [Min, Max]: 2.00 [1.00, 7.00]	Median [Min, Max]: 15.0 [10.0, 15.0]	Median [Min, Max]: 16.0 [10.0, 19.0]	Median [Min, Max]: -1.00 [-6.00, 4.00]
		Missing: 1 (0.3%)	Missing: 1 (0.3%)	Missing: 1 (0.3%)

In the table is indicated the speciality and the Swedish name of the hospital building from which are referred the metrics. Supplementary Table 2 reports the same information for all the hospital wards.

There was a pattern of hospital admission for older patients (Table 2). This correlation with hospital admission explains why also this variable was relevant for the length of stay regression. More than half of these elderly admitted patients arrived by ambulance. Furthermore, patients with generic symptoms had a huge impact on the hospital admissions for all the wards (Fig. 5, Supplementary Fig. 1, and Supplementary Table 2). The Sankey flow in Fig. 5 shows that these patients have been admitted across wards in the hospital, the high clinical variability of the data is also reflected in the ED process abstraction. This aspect was pointed out by the spider-net obtained from the ED-hospital wards pathways extracted by applying a direct-to-follow graph process mining algorithm (Supplementary Fig. 3). In detail, Supplementary Table 2 underline that surgery ward pressure was mainly from patients with abdominal pain, cardiology by chest pain, and acute medicine by potential high fragile geriatric patients (difficulty of breathing). Supplementary Fig. 4 shows that misallocated patients were admitted everywhere in the hospital (17.6% of the total records, Supplementary Table 3). This phenomenon was more common during the year were neuro, thorax, “ear, nose and throat”, genecology, and “plastic and maxillofacial surgery” (Supplementary Table 4).

### Patients re-visiting ED: a global resonant pressure

Figure 2 shows that patients that re-visited the ED impacted significantly to all the KPIs during the entire year (33% ambulance, 35.5% scans, 29.1% hospital admissions on the total yearly visits). We detected few cases of patients that revisited the ED more than 10 times (n = 96, from which the max number of re-visits for a single patient was 65), but from which the cumulative effect with the visits of the other patients across the year was resonantly impacting the ED sources. The analysis of the concomitant chief complaint and ICD10 subsequently occurred after each re-visit showed what were the typical profiles of these patients (Supplementary Table 5). We detected three main patterns: patients having subsequent generic symptoms before receiving a specific diagnosis after several re-visits (e.g., such as consecutive visits with abdominal pain R104 before ileus K590 being diagnosed), patients having psychological issues with consecutive cases of injuries by self-inflicting damage or poisoning, and highly fragile older patients that need basic care (e.g., general weakness or constipation).

As mentioned before, clinical experts were already aware about the importance of solving the issue of geriatric patients. The geriatric flow is characterized by both those residing in Uppsala’s geriatric facilities and those living independently at home. These individuals, often highly fragile and requiring basic care, presented a unique challenge, particularly for those living at home, where logistical difficulties in the discharge process frequently led to prolonged lengths of stay, exceedingly more than three days. From the data was not possible to clearly detect the geriatric patients not living in the special facilities, even with the aid of the clinical experts, because of the similar characteristics with patients with non-specific diagnosis.

The analysis provided further information regarding the impact of re-visits on the ED, thus also underlining the competitive management of the other sub flows. Re-visiting patients with psychological profiles were recognized by the clinicians as a known issue for the ED. Instead for what concerns the delay of specific diagnoses, the data information was not sufficient to detect and stratify these patients in more precise sub-flows, still underlying the impact of this bulk of patients.

## Discussion

In this paper we designed a comprehensive pipeline to analyse healthcare production data following ED patient flows aimed to leverage real-world data potentiality to study the overcrowding phenomena. The approach showed in Fig. 1 was designed to account the real-world data challenges in all the steps of the analysis with the involvement of clinical experts, thus allowing to overcome the limitations of the data and explore overcrowding of the Uppsala University Hospital ED from a whole system perspective. According to the knowledge of the authors, this is the first study of ED flows using healthcare production data with this wide a large overview regarding data information, processes, and interaction with hospital wards and external processes.

In traditional data-driven approaches, clinical experts are usually involved in the final step where outcomes are discussed. The involvement of clinical experts in all steps of the pipeline (Fig. 1, steps a-c) was fundamental to contextualise the data with medical and operational knowledge, and informing the analysis and the findings for a proper discussion on how to solve the overcrowding of the ED. This approach underlined the gap that there is between data records and actual operations and how decision-making reasoning is difficult to integrate with the data.

From the multi-objective analysis (Fig. 1, steps b-c) it emerged that there were multiple sources that led to the ED overcrowding. These rely on both clinical and organisational factors and are connected to internal and external processes of the ED environment. This is a result we would expect because it is well-known that the management of overcrowding in EDs is a complex multi-constrained problem due to the interaction between logistic and clinical aspects^16,17,34^.

In detail, the results discussed in Table 2 revealed that the main sources of the ED saturation were connected to the high number of patients classified as non-urgent with generic symptoms, the delayed specific diagnosis and hospital admission decision from which multiple imaging evaluation was required, the delayed admission to the hospital because of the lack of available beds in the wards, and the external pressure of high frequent re-visits of geriatric, psychiatric and patients with subsequent generic symptoms before receiving a specific diagnosis.

The aggregated analysis of the outcomes (Fig. 1 steps c.1) allowed to estimate the magnitude of causes of the overcrowding known a priori (e.g., patients seeking basic care and the geriatric flow) and reveal novel insights (e.g., the global impact of the cumulative re-visits). The limitation of the data information emerged when it was not possible to define well separated sub-flows from the clinical variables even with the clinical feedback.

The retrospective evaluation (Fig. 1, steps c.2) provided hints regarding aspects to focus on the future for improving the understanding of overcrowding and explore key strategies.

For what concern the internal improvement of ED operations, a key aspect to discuss will be how to make the evaluation process faster and more accurate of patients with non-urgent need of care but that are difficult to evaluate. These were the ones with delayed decisions regarding discharge or hospital admissions requiring several imaging evaluations, and the ones visiting frequently the ED with generic diagnosis before receiving a specific one. Another internal aspect to discuss regards the improvement of the collected data information that can be re-utilise for future analysis.

There is the need for a deeper discussion regarding the efficacy of the primary care systems outside the hospital. The ED pressure would be drastically decreased if patients could seek basic or non-urgent care outside the ED (e.g., geriatric flow, green triage, or patients with feared health complaint). Furthermore, a deeper study regarding the overcrowding of hospital wards and the management of highly frequent visits of psychiatric patients would be beneficial for the ED distress.

The proposed approach allowed to study concomitantly multiple components of emergency flows and several KPIs, including considerations to where patients are admitted and if these will re-visit the ED. This allowed to overcome the previous limitations of studies that focused merely on specific flows or singular KPIs, especially for what concern analysis of throughput interventions with lack of considerations regarding inflows and outflows^60^. Furthermore, our approach connected considerations regarding the volume of flows with their clinical variability, thus enriching insights of previous analysis where these components were considered separately^16,33,36,51,63–65,73^.

Our approach demonstrated the key role clinical expert’s involvement in data-driven approaches for improving the understanding of overcrowding. This aspect of the pipeline allowed to leverage the gap between data and clinical processes and explore the gap between the collected data and the practical utility^35^. So far, the utilization of real-world data has been focused more on the operational management rather than discussion about the healthcare policies^41,61^, and it is well known that there is a lack of qualitative approaches to healthcare problems^74^.

In data-driven approaches, the widely recognized principle of 'garbage-in garbage-out' cautions against relying on insufficient or unreliable data to solve complex tasks. However, when it comes to use data for addressing real-world healthcare challenges, this paradigm should not be seen as a disruptive barrier, but as an occasion to discuss how to improve and leverage collected information and how this could provide insights for the improvement of the system operations.

From our whole-system analysis, it emerges that pure data-driven approaches would not be a definitive solution for analysing ED overcrowding. In contrast, this paper shows that by adopting an inclusive approach, not only can we enhance real-world data potential to improve operational decisions within the emergency department, but it also provides an opportunity to facilitate policy-making discussions that encompass broader aspects affecting the healthcare system, such as engagement with local municipal or regional authorities. For example, from our results it emerged that an improvement of geriatric and psychiatric pathways, and a serious discussion regarding primary care delivery, would be crucial to decrease pressure on the ED.

From the obtained results, ED resources appear to be squeezed from all directions, from the primary healthcare delivery to the overcrowding of hospital wards that impact on the ED admission process with the boarding. Finally, we can detect the origin of the possible solutions by analysing this mismatch between community needs and the delivery of care from the whole-system perspective not isolating only emergency medicine. The take home message is that we should learn beyond the pure empirical approaches by involving clinicians and managers, and from there we can start to design future solutions to the ED overcrowding looking beyond the walls of the ED and the hospital.

Despite the significance of our work, there are certain limitations that should be acknowledged. Firstly, the study was conducted at a single centre in Uppsala, Sweden, which may limit the generalizability of the findings to other healthcare settings. Furthermore, the analysis was based on data from a one-year time window, which may not fully capture long-term trends and variations in the ED workflow. It is relevant to note that the available data lacked detailed clinical variables, such as blood test results, and the level of granularity regarding the decision-making process by clinicians was limited. This relied on the fact that the analysed records were health care production data. This enrichment of the data information would be beneficial for the improvement of multivariate regression models for the length of stay since the current information is confounded by the saturation of the system.

Moreover, the discussion and expert input primarily involved clinical practitioners, and no other stakeholders and actors in the healthcare system. The absence of comprehensive discussions with external stakeholders, such as policymakers, administrators, and patients, may have limited the breadth of insights and potential solutions generated from the analysis.

It is essential to recognize these limitations as they highlight the need for future research to address these gaps. This could include conducting multi-centre studies to validate the findings across different healthcare contexts, extending the time window of analysis to capture long-term dynamics, and enhancing data collection efforts to include more detailed clinical variables. Additionally, engaging a broader range of stakeholders in the analysis and decision-making process can lead to more comprehensive and impactful strategies for addressing the challenges faced by EDs and improving overall healthcare delivery.

As mentioned before, overcrowding in EDs is an international problem^1,6^, and that regardless the massive quantity of works aimed to operational research there is still a lot of work to do to solve this problem^21,22^, especially in the current discussion on real-world evidence and healthcare data^19,26,75^. In the current discussion regarding data-driven healthcare in international settings, our pipeline could be interesting to implement for participatory approaches and to facilitate discussions about the problem from the perspectives of different healthcare policies.

## Conclusions

Our analysis reveals insights into ED overcrowding and enables to identify systemic issues and directions for solutions. The whole systems perspective opened the scope to the boundary effects of inflow and outflow of the ED inside the hospital. Finally, our approach demonstrates that to enhance and unlock the potential of real-world data in studying ED overcrowding challenge we need to look to systems beyond the walls of the ED and the hospitals to solve this problem.

### Supplementary Information

Supplementary Information 1.

Supplementary Tables.

## Data Availability

The dataset used and analyzed during the current study and computer code are available from the corresponding author on reasonable request.
