# Supplementary material for: Diagnosing an overcrowded emergency department from its Electronic Health Records
Source: Sci Rep. 2024 Apr 30;14:9955. doi: 10.1038/s41598-024-60888-9 (PMC11061188; doi:10.1038/s41598-024-60888-9)
Supplement: Supplementary file 1 — Supplementary Information 1. [file 41598_2024_60888_MOESM1_ESM.docx]

SUPPLEMENTARY MATERIALS: Diagnosing an overcrowded emergency department from its Electronic Health Records

Marzano Luca*^1^, Darwich Adam S.^1^, Raghothama Jayanth^1^, Lethvall Sven^2^, Falk Nina^2^, Bodeby Patrik^2^, Meijer Sebastiaan^1^

1. Department of Biomedical Engineering and Health Systems, KTH Royal Institute of Technology, Stockholm, Sweden
2. Uppsala University Hospital, Uppsala, Sweden

**Corresponding author: Luca Marzano, Department of Biomedical Engineering and Health Systems, KTH Royal Institute of Technology, Stockholm, Sweden, email: lmarzano@kth.se*

## **Abstract**

In this document we provided additional materials to the manuscripts “How to diagnose an overcrowded emergency department from its EHRs? Enhancing opportunities and challenges of real-world data from a whole-system perspective”

# **Supplementary Figures**


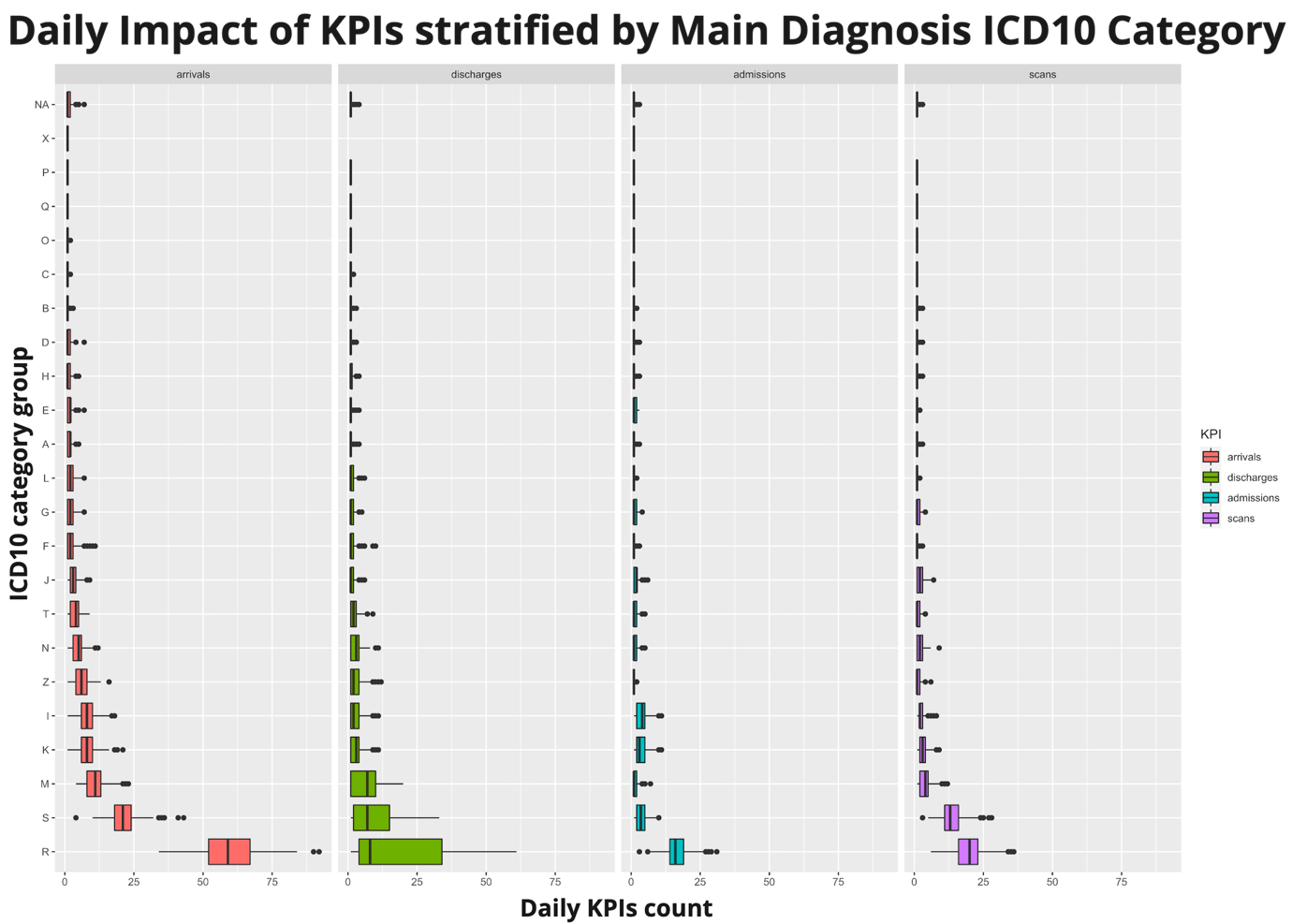


Supplementary Figure 1. Daily measures of the KPIs in the ED stratified by main ICD10 category groups.


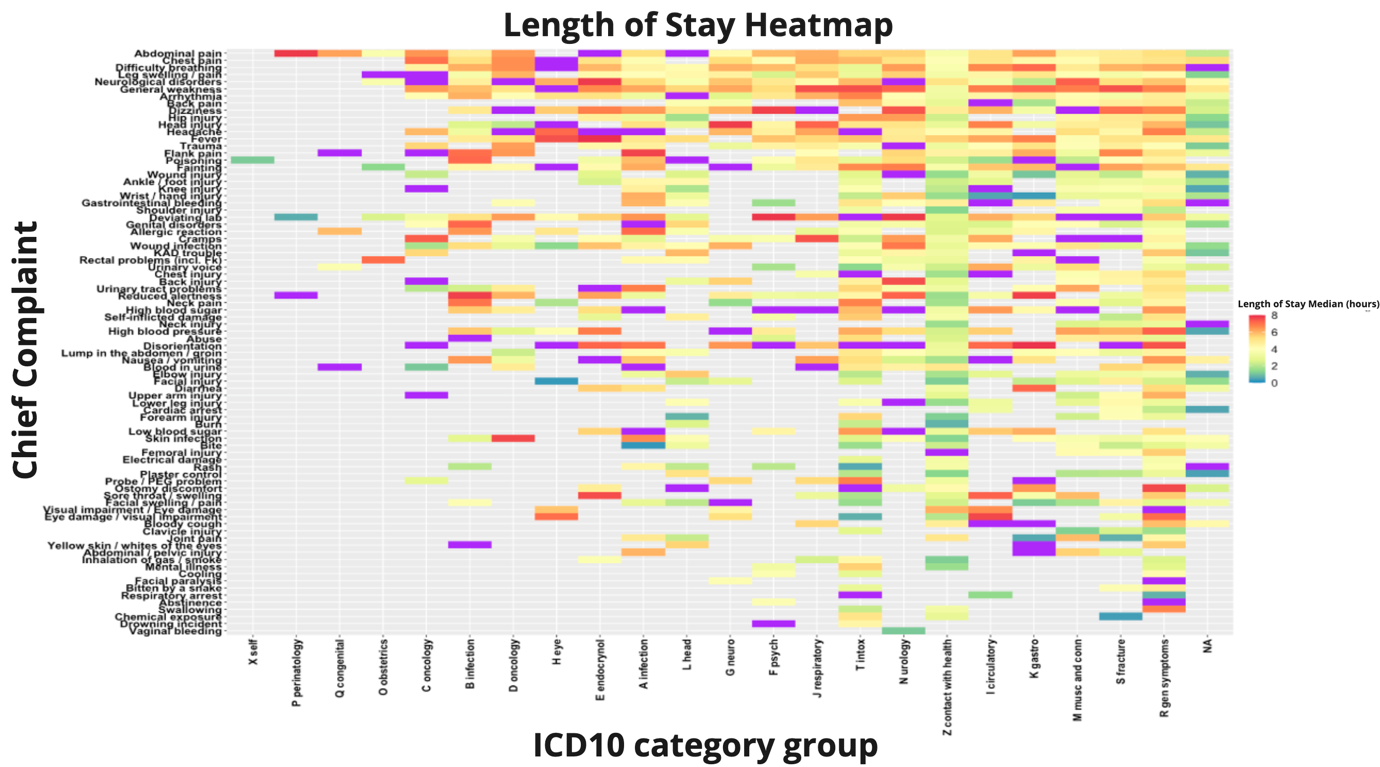


Supplementary Figure 2. Heatmap of length of stay in function of ICD10 diagnosis and chief complaint. Measures longer than 8 hours were marked with purple.


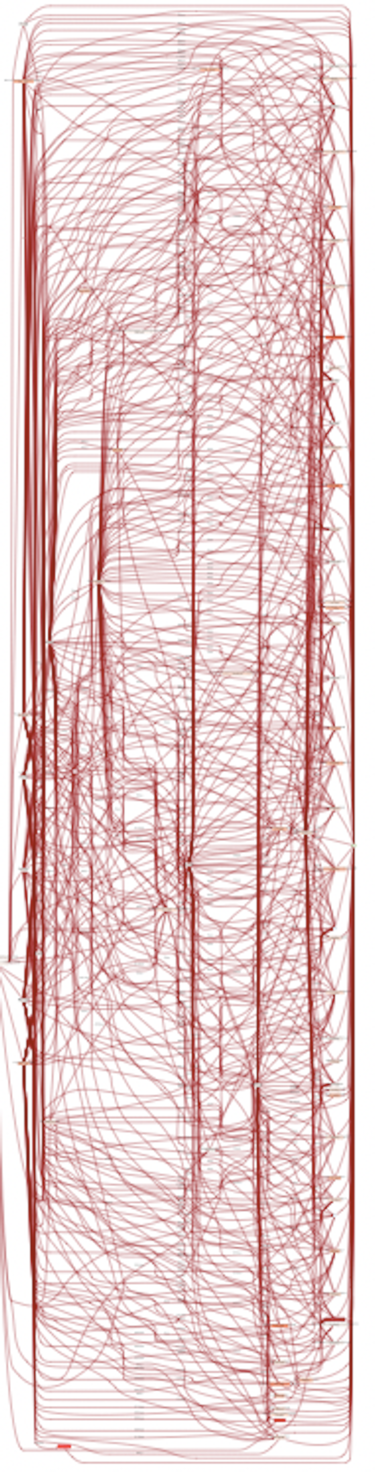


Supplementary Figure 3. Pathways of ED patients in the hospital wards. Graph obtained with a standard graph-to-follow process mining algorithm.


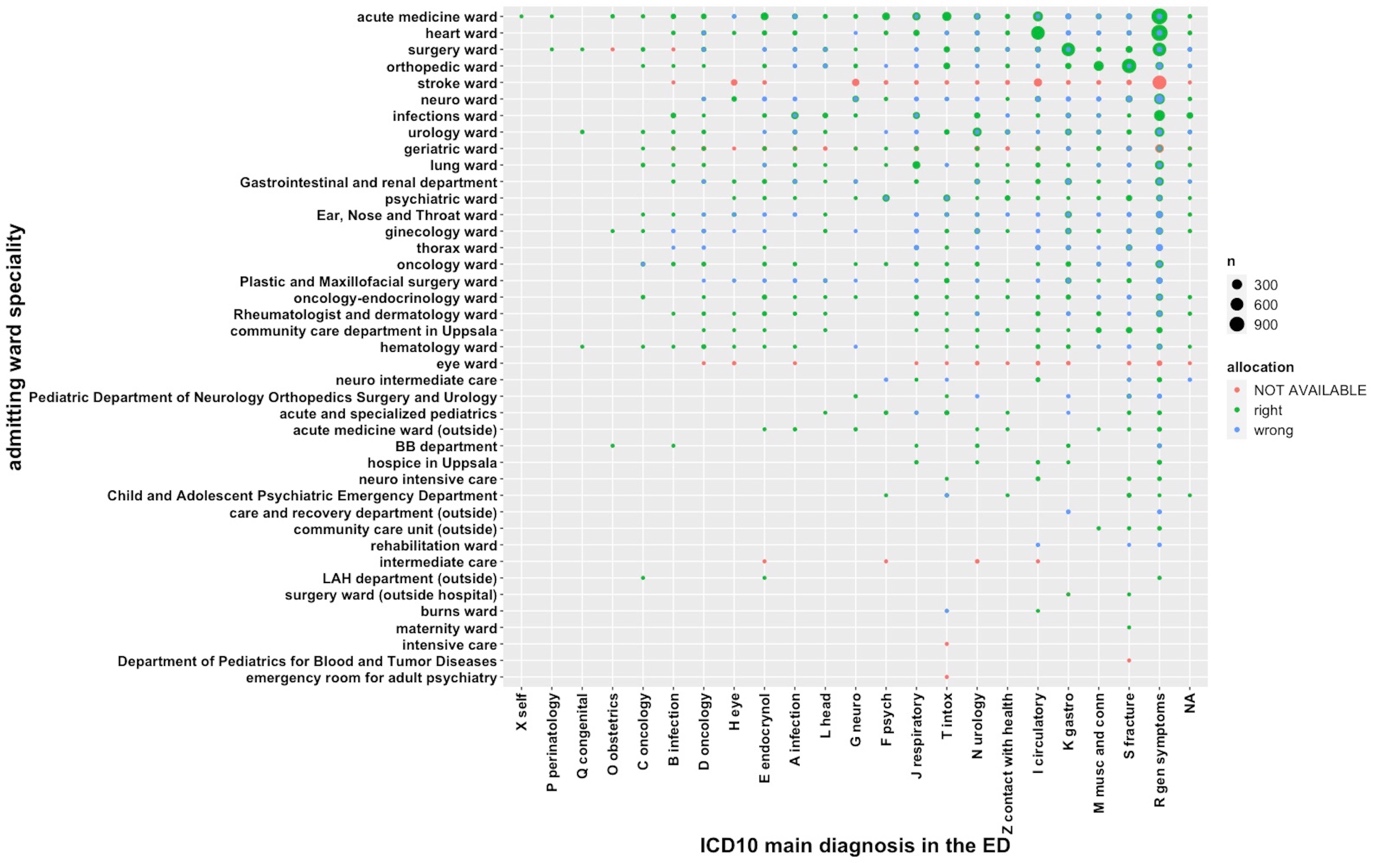


Supplementary Figure 4. Right and wrong allocation map after admission to hospital wards in function of the diagnosis made in the ED.

# **Supplementary Tables**

Supplementary Tables 1-5 are reported in the table sheets attached to the Excel file included during the submission.

# **Supplementary Results**

A multivariate regression was performed to assess the relationship between length of stay and the variables (age, sex, ambulance, chief complaint, triage, scan, ICD10 main diagnosis category, Medical Alarm unit, and discharge method). Regression was performed in R using the lm() stats function.

The multivariate regression confirmed the high impact of scans on length of stay (Figure 4) and detected as relevant the reason for discharge and the age. Some variables with a slight shorter median of the length of stay in Figure 4 showed a less but relevant contribute in the regression (triage: “red”, ICD10 category: “S fractures”, and ICD10 category “Z feared complaint”).

However, the R-square coefficient underlined that the linear assumption for length of stay was not captured by the data (R: 0.26). This is a confirmation that the saturation of the ED reflected in the data makes challenging an effective multivariate prediction of the length of stay from the data.

Supplementary Table 6. Most relevant variables in the multivariate regression of the length of stay. Output of the lm() function on R stats related to the estimated linear coefficient, standard error, and statistical relevance.

| Variable | Coeff. Estimate | Std. Error | p-value |
| --- | --- | --- | --- |
| Age | 0.0129261 | 0.0009368 | < 2e-16 |
| Scan (Yes) | 3.1270689 | 0.0410533 | < 2e-16 |
| Reason For Discharge (Home) | -0.7800265 | 0.0646935 | < 2e-16 |
| Reason For Discharge (Hospital Admission) | 1.2432695 | 0.0689589 | < 2e-16 |
